# M. Faye’s Theory of the Crust of the Earth

**Published:** 1881-04

**Authors:** 


### M. FAYE'S THEORY OF THE CRUST OF THE EARTH.

M. Faye has propounded a new theory of the internal structure of the earth, an important feature of which is that its solid crust is much thicker under the seas than under the continental masses. The oscillations of the pendulum and the direction of the plumb-line are known to be subject to variations in consequence of the neighborhood of a mountain, or even of a hill, calculations based upon which enabled Maskelyne to determine the density of the globe. When, however, experiments with the plumb-line and pendulum were applied to table-lands and to the grander mountain-ranges, the deviations corresponding to the magnitude of the masses which were expected were not shown. The pendulum which is sensitive to the presence of the Great Pyramid of Egypt gives no sign of the neighborhood of the Himalayas. Further than this, a real deficiency of attraction has been observed upon continents, as if there were a great hollow under them; and the failure of mountain-masses to deflect the pendulum has been actually attributed to the existence of cavities in them. On the other hand, when the investigation is transferred to the sea, the weight is found to be too great, and is in excess of what is demanded by theory, as evidently it falls short of it on continents. Hence, if we suppose that there is a lack of matter under the continents, we must also suppose that there is under the seas an accumulation of it above the average for the whole earth. M. Faye suggests, to account for these contradictions, that the cooling of the earth is going on faster and has taken place to a greater depth under the oceans than under the continents. The temperature at 12,000 feet of depth below the sea is a little higher than the freezing-point; at the same depth under the continental masses it is computed to be about 300°. The matter is kept at this temperature by the superior strata of earth almost impermeable to heat, and through which the heat that actually escapes is hardly perceptible. The crust of the earth in such a situation can increase in thickness only at the slowest. Under the sea, on the other hand, matter at the same depth is in almost immediate communication with a cold of the freezing point, and, instead of having some non-conducting strata above it to prevent its escape, the heat is immediately absorbed in a cold of polar intensity. A similar difference exists deep in the beds of the submarine rocks, for the water is imbibed in their pores to a greater depth than in the sub-continental rocks, and the heat is conveyed away from them by the vertical convection of the warmed water rising in them. The more ancient the existing beds of the sea, the greater is the thickness of crust that supports them as compared with that of the continents.

—*Popular Science Monthly.*

